# Inhibition of c-FLIP alongside TRAIL treatment suppresses prostate cancer stem cell activity

**DOI:** 10.1038/s41416-026-03359-4

**Published:** 2026-03-03

**Authors:** Daniel J. Turnham, Rhiannon French, Fiona M. Frame, Valerie S. Meniel, Norman J. Maitland, Richard W. E. Clarkson

**Affiliations:** 1 European Cancer Stem Cell Research Institute, Cardiff University, Hadyn Ellis Building, Maindy Road, Cathays, Cardiff, CF24 4HQ UK; 2 Centre for Biomedical Research, School of Applied Sciences, University of the West of England, Coldharbour Lane, Frenchay, Bristol, BS16 1QY UK; 3 Cancer Research Unit, Department of Biology, University of York, Heslington, YO10 5DD UK

**Keywords:** Prostate cancer, Cancer stem cells, Targeted therapies

## Abstract

**Background:**

Prostate cancer is a leading cause of cancer-associated death in men worldwide. Inhibition of the Cellular FLICE-like Inhibitory Protein (cFLIP), which is overexpressed in prostate cancer, alongside TRAIL treatment can trigger apoptosis and suppress cancer stem cell (CSC) activity in different cancer types but has not been fully explored in prostate cancer.

**Methods:**

Established and primary prostate cancer lines were treated with the cFLIP inhibitor, OH14, in combination with recombinant TRAIL to investigate changes in viability and colony forming potential. Patient-derived xenograft (PDX) tumour cells were treated ex vivo and re-transplanted into mice in limiting dilution assays. Docetaxel resistant PC-3 cells were also treated with OH14 +/- docetaxel, while PDX tumours were treated in vivo with this combination.

**Results:**

Combined OH14 and TRAIL treatment induced a potent apoptotic response in prostate cancer cells, significantly reducing viability and CSC activity compared to single agents. OH14 also sensitised tumour cells to docetaxel both in vitro and in vivo.

**Conclusions:**

Inhibition of cFLIP in combination with either TRAIL or docetaxel has the potential to be used as a novel therapeutic approach to provide more potent, long-lasting benefits to men with prostate cancer.

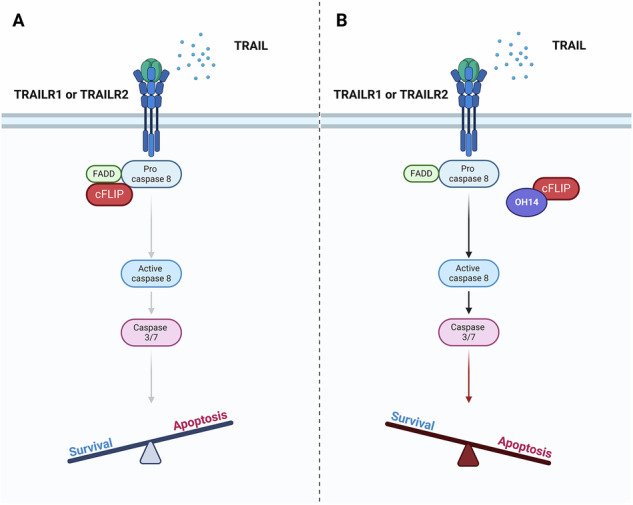

## Background

Prostate cancer is the second most commonly diagnosed cancer in men worldwide and kills over 375,000 men annually making it the fifth leading cause of death by cancer in men [1]. Although the 5 year survival rates for men with localised disease is almost 100%, for those with more aggressive tumours that have spread beyond the prostate and/or have lost sensitivity to hormone therapy the prognosis is far worse [2, 3]. Androgen deprivation therapy (ADT) is commonly used to control hormone-sensitive prostate cancer (HSPC); however, resistance eventually occurs leading to the development of aggressive, hard to treat castration-resistant prostate cancer (CRPC). For patients with CRPC, survival is often measured in months rather than years, with current therapies including taxane-based chemotherapy, enzalutamide, abiraterone and radium-223 (for bone metastases) no longer curative [4, 5]. Improving clinical outcomes for patients with CRPC through the development of novel therapeutic approaches remains an important clinical challenge.

The Cellular FLICE-like Inhibitory Protein (cFLIP) is an important component of the extrinsic apoptosis pathway and is overexpressed in several cancer types, including prostate cancer where protein expression is upregulated in CRPC [6]. The extrinsic apoptosis pathway is activated by ligand binding to cell surface death receptors, which triggers the formation of a death-inducing signalling complex (DISC) and downstream caspase activation, however cFLIP can disrupt this process by inhibiting caspase-8 activation [7, 8]. Both long and short isoforms of cFLIP exist (cFLIPL and cFLIPs) which each have two Death Effector Domains (DEDs) that are crucial for their interaction with other members of the DISC [9]. cFLIPL is predominately thought to inhibit apoptosis by competing with procaspase-8 for binding to FADD at the DISC, while cFLIPs can form heterodimers with procaspase-8, preventing it’s subsequent dimerization and activation [10]. Tumour Necrosis Factor Alpha Receptor Apoptosis Inducing Ligand (TRAIL), is a well characterised activator of the extrinsic pathway and has been studied extensively as a therapeutic agent since it was shown to selectively trigger cell death in tumour cells [11]. Despite initial preclinical promise, TRAIL has failed to demonstrate significant therapeutic efficacy in several clinical trials, with resistance to TRAIL-mediated apoptosis through the expression of cFLIP cited as one potential contributor [12, 13].

Genetic silencing of cFLIP expression has been demonstrated to induce spontaneous apoptosis-mediated cell death without the need for ligand-mediated activation of the extrinsic apoptosis pathway, while cFLIP inhibition has also been shown to sensitize prostate cancer cell lines to TRAIL-induced apoptosis [6, 13–15]. Interestingly, despite data to suggest that silencing both long and short cFLIP isoforms individually can sensitise prostate cancer cell lines to TRAIL, in cells with acquired resistance to TRAIL following prolonged exposure, only the former can maintain this sensitisation [13, 16]. Furthermore, cFLIP suppression can sensitise prostate cancers to both bicalutamide and docetaxel, suggesting that cFLIP inhibition could potentiate the effects of clinically relevant CRPC treatments [6, 14]. Interestingly, the combination of cFLIP inhibition and TRAIL has been demonstrated to specifically-target cancer stem cells (CSCs) in breast cancer and therefore has the dual capability to shrink both tumour mass through apoptosis-mediated cell death and inhibit tumour relapse and metastasis by targeting the small population of CSCs that are widely regarded as being responsible for these processes [17, 18]. The recent development of small molecule modulators of cFLIP, that either disrupt or stabilise cFLIP/caspase 8 interactions at the DISC to de-repress TRAIL mediated apoptosis, offer a proof of principle for the potential of pharmacological modulators of cFLIP in a clinical setting [19**–**21].

In this study, we use OH14, a cFLIP inhibitor that binds to a unique pocket within the DED1 domain of cFLIP to disrupt it’s incorporation into the DISC and thereby permitting procaspase-8 cleavage and activation following engagement of the extrinsic apoptosis pathway [19]. OH14 is used to demonstrate how inhibition of cFLIP in combination with TRAIL treatment can sensitize prostate cancers to TRAIL-mediated apoptosis and can reduce CSC activity in both established and primary prostate cancer cells in vitro. In addition, we show that OH14 treatment alone both in vitro and in vivo can improve the efficacy of docetaxel treatment to inhibit prostate tumour growth, which offers new opportunities in the future for treatment of patients with late-stage prostate cancer.

## Methods

### Cell lines and reagents

Established prostate cancer LNCaP and PC-3M cell lines (obtained from Professor Norman Maitland’s laboratory at the University of York) were cultured in RPMI 1640 GlutaMAX media (Thermo Fisher Scientific, Cheshire, UK), supplemented with 10% foetal bovine serum (Thermo Fisher Scientific) and 1% penicillin-streptomycin (Thermo Fisher Scientific) at 37 °C in 5% CO_2_. Docetaxel resistant PC-3 cells (PC-3 D12) and age matched control PC-3 cells (PC-3 Ag) were kindly provided by Professor William Watson, University College Dublin, following previously established cell culture conditions [22]. All cell lines were regularly tested for mycoplasma contamination.

OH14 was synthesised in the School of Pharmacy and Pharmaceutical Sciences, Cardiff University as previously described [19], with cells treated at 100 µM in vitro. Recombinant soluble human TRAIL was purchased as super-killer TRAIL (Enzo Life Sciences, NY, USA) with cells treated at 20 ng/ml. The pan-caspase inhibitor, Z-VAD-FMK (R&D systems, Abingdon, UK), was used at a concentration of 20 μM and docetaxel (Generon, Slough, UK) was used at 1 nM. ON-Target plus SMART pool siRNAs (Dharmacon, Cambridge, UK) targeting CFLIP (#L-003772-00-0005) or non-targeting control siRNAs (#D-001810-10) were transfected on adherent cells using lipofectamine RNAiMax (Thermo Fisher Scientific) and serum-free Opti-MEM (Thermo Fisher Scientific) in accordance with the manufacturer’s instructions.

### Primary tissue culture

Patient samples were collected with ethical permission from Castle Hill Hospital (Cottingham, Hull) (Ethics Number: 07/H1304/121) and through the Wales Cancer Biobank which is funded by Health and Care Research Wales (Project Number: 16007) [23]. Use of patient tissue was approved by the local research ethics committees. Patients gave informed consent and all patient samples were anonymized. Other investigators may have received specimens from the same subjects. Primary cell cultures were derived from consenting patients undergoing routine radical prostatectomy and channel transurethral resection (TURP) surgery, with tumour grade confirmed by histologic examination of representative fragments by a uropathologist. Epithelial cells were isolated and cultured with Mitomycin C (Abcam, Cambridge, UK) treated STO (mouse embryonic fibroblast) feeder cells following previously published protocols [24]. Viability assays were performed on confluent epithelial cultures while colony formation assays were performed in the presence of STO feeder cells. All data shown represents samples that were tested 3 times independently.

### Apoptosis and viability assays

Real-time apoptosis assays were performed using the Incucyte® S3 Live-Cell Analysis System (Essen Bioscience, Hertfordshire, UK). Cells were grown in 96-well plates and supplemented with Annexin V green reagent (Essen Bioscience) at the time of siRNA transfection, which emit a green, fluorescent signal upon binding to apoptotic cells. Cells were maintained within the Incucyte system for the duration of experiments and Annexin V staining analysed using the Incucyte live-cell-imaging software to detect positive-fluorescent signals.

To determine end-point cell viability Cell Titre Blue assay (Promega, Southampton, UK) was used on cells grown in 96-well plates. On the day of analysis, 20 µl of Cell Titre Blue reagent (Promega) was added to 100 µl of growth medium in each well. After 1 h of incubation at 37°C in 5% CO_2_ fluorescence intensity was measured at 560/590 nm using a ClarioStar plate reader (BMG Labtech, Aylesbury, UK). All data shown represents a minimum of 3 biological replicates with additional replicates indicated by the individual data plots.

### Colony formation assay

Following treatment, viable cells were determined by trypan blue (Thermo Fisher Scientific) seeded at a density of 250 cells/well in a 12-well plate format and cultured for 7-10 days. To stain colonies, culture medium was removed, and cells washed once with PBS before crystal violet solution (Sigma, Welwyn Garden City, UK) was applied to wells and incubated for 15 mins at room temperature. Solution was removed and wells were rinsed under running tap water. Colony plates were imaged using a GelCount plate reader (Oxford Optronix, Oxford, UK) and colonies containing approximately 20 or more cells were manually counted using ImageJ software [25]. All data shown represents a minimum of 3 biological replicates, with primary data demonstrating individual samples that were replicated 3 times independently.

### qRT-PCR

RNA was extracted using an RNeasy mini kit (Qiagen, Manchester, UK) following manufacturer’s instructions, before cDNA was generated using a High-Capacity cDNA Reverse Transcription Kit (Thermo Fisher Scientific). A QuantStudio 7 Real-Time PCR machine (Applied Biosystems, Altrincham, UK) was used for multiplexed qRT-PCR reactions containing template cDNA, TaqMan Universal Master Mix II (Thermo Fisher Scientific) and predesigned TaqMan primers targeting *CFLAR* (Hs00153439) and ACTB (Hs99999903_m1). All data shown represents 3 biological replicates.

### Animal studies

All mouse experiments were performed at Cardiff University in accordance with the Animals (Scientific Procedures) Act 1986 guidelines and approved by the UK Home Office under project licence PPL 30/2849. All experiments were performed on male mice aged between 6-8 weeks, that were housed in specific pathogen-free facilities under a 12-hour light/dark cycle and controlled temperature (20–25 °C). Mice were kept in cohorts of 2-6 mice per cage enriched with nesting materials and chew sticks. Experimental treatment groups were not blinded throughout each study.

For limiting dilution assays, the H455 patient-derived xenograft (PDX) model was used, with preliminary studies (data not shown) performed to determine the tumour growth rate at different seeding densities and to establish the sample size required. This PDX was previously derived from a 67-year-old CRPC patient that had previously received hormone therapy [26]. Frozen tumour pieces were derived in NOD-SCID-Gamma mice (Charles River, Kent, UK), supplemented with 35 mg/kg 5-α-DHT diet (Ssniff, Soest, Germany). Once tumours reached ethical limits mice were sacrificed (by cervical dislocation) and the tumours were harvested and collected in transport media DMEM/F12 supplemented with 10% FBS, 1% pen/strep and Glutamax (Thermo Fisher Scientific). Tumours were then processed as previously described [24] and instead of being plated for culture, cells were suspended in Ficoll (Sigma) and centrifuged at 1800rpm for 30 min. Live cells were collected from the opaque interphase layer with a Pasteur pipette, mixed with D10 media (transport media minus antibiotics) and centrifuged to pellet cells. Cells were washed once with MACS buffer (PBS/2 mM EDTA/0.5% bovine serum albumin) and any remaining mouse cells removed using the mouse cell depletion kit (Miltenyi Biotec, Surrey, UK) and LS magnetic separation columns (Miltenyi Biotec) following manufacturer’s instructions. The collected cells were then treated suspended in D10 media containing DMSO (control group), OH14 (100 µM), TRAIL (20 ng/ml) or OH14 and TRAIL combination for 24 hours on a MACS rotator at 37°C in 5% CO_2_. Following treatment, viable cells were counted using Trypan Blue and re-transplanted subcutaneously in 100 µL of a 50:50 PBS and Matrigel (Corning) mix bilaterally in serial dilutions into NSG mice assigned using simple randomization (total tumours analysed per treatment: DMSO n = 34, OH14 n = 33, TRAIL n = 32, combination n = 28). Mice were maintained on androgen supplemented diet, distributed between cages at random to minimise potential confounders and sacrificed once both tumours were palpable or after 3 months post tumour transplantation and counted as negative if no palpable tumour was detected.

For the CRPC xenograft treatment study, H445 PDX tumours were grown bilaterally until tumours reached 20-100mm^3^, as determined by calliper measurements and the modified ellipsoidal formula (V = ½ (Length × Width^2^). At this point mice were randomly assigned treatment groups in blocks to ensure each treatment group was equally distributed, with the required tumour numbers calculated based previous studies utilising OH14 in other cancer types (total tumours analysed per treatment: control n = 9, OH14 n = 8, TRAIL n = 8, combination n = 8). Control mice were treated with vehicle (2.5% DMSO in sterile water) and experimental mice were treated with OH14 (10 mg/kg), both delivered via i.p daily, 5 days a week. Docetaxel (10 mg/kg) was given i.p once a week, with mice treated in no systematic order. Mice were monitored daily and weighed regularly, with any mice showing signs of sickness or weight loss of over 10% of starting weight culled and not used in the analysis. Average tumour size was monitored as the primary outcome and once control tumours approached ethical limits all mice were sacrificed.

### Bioinformatics analysis

The Prostate Cancer Atlas was used to investigate RNA-seq datasets from normal prostate and prostate cancer samples from different molecular subtypes [27]. Additional TCGA analysis was performed using UALCAN software [28, 29].

### Statistical analysis

To determine significance, statistical analysis was performed on GraphPad Prism 10 software, which was also used to determine if data was normally distributed and applies appropriate tests or corrections if variance assumptions are not met. For single comparisons an unpaired two-tailed students t test was performed. For multiple comparisons a One-Way ANOVA was used for endpoint assays and a TWO-way ANOVA was used for time-course experiments, both with Tukey correction. For limiting dilution assays Extreme Limiting Dilution Analysis (ELDA) software was used to estimate the frequency of CSCs [30].

## Results

### OH14 mimics cFLIP silencing and sensitises prostate cancer cell lines to TRAIL-mediated apoptosis

Genetic silencing of cFLIP using siRNA has previously been shown to spontaneously induce cell death in prostate cancer cells, while it is also able to sensitize prostate cancer cell lines to TRAIL-mediated killing [6, 13–15]. In breast cancer cell lines, OH14, a small molecule cFLIP inhibitor has been shown to replicate the effects of cFLIP silencing to induce apoptosis in combination with TRAIL [19].

**Fig. 1:**
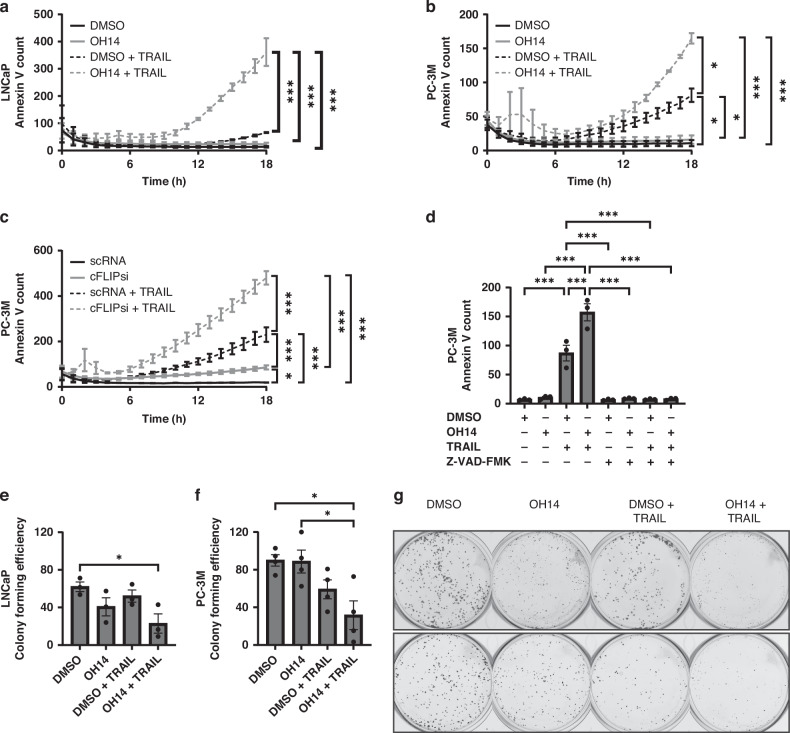
Investigating the response of prostate cancer cell lines to OH14 and TRAIL. **a** LNCaP and **b** PC-3M prostate cancer cell lines were pretreated with OH14 (100 µM) for 1 h followed by TRAIL (20 ng/ml) for an additional 18 h with changes in Annexin V counts over the 18 h TRAIL treatment period analysed using Incucyte® real-time software. **c** PC-3M cells were treated with cFLIP targeting siRNA (cFLIPsi) or non-targeting control siRNA (scRNA) for 24 h followed by TRAIL treatment for 18 h before Annexin V counts were assessed as previously described. **d** PC-3M cells were treated with OH14 in addition to a pan-caspase inhibitor (Z-VAD-FMK, 20 µM) for 1 h followed by 18 h TRAIL treatment with Annexin V Incucyte counts at the end of treatment plotted to determine apoptosis. Following OH14 and TRAIL treatment in (**e**) LNCaP and (**f**) PC-3M cells as before, viable cells were counted and seeded at low density into colony formation assays. Colonies were manually counted and colony forming efficiency was plotted as a percentage of the number of cells initially seeded. **g** Representative images of colony forming assays in LNCaP and PC-3M cells. Each data point represents a biological repeat, while Annexin V assays represent the average of 3 independent repeats with error bars shown as SEM of all data points. *p < 0.05, **p < 0.01, ***p < 0.001, One-way ANOVA for endpoint comparisons or Two-way ANOVA for time course Incucyte assays, both with Tukey correction for multiple comparisons.

To test whether OH14 may be able to replicate this effect in prostate cancer, TRAIL resistant LNCaP and TRAIL sensitive PC-3M cell lines were assessed following treatment with OH14 alone and in combination with recombinant TRAIL [13]. Each cell line was pretreated with OH14 for 1 h before TRAIL was added for an additional 18 h of treatment, with changes in apoptosis assessed using real-time Annexin V assays. OH14 alone had no effect on apoptosis, however it did sensitise each cell line to TRAIL-mediated apoptosis, demonstrated by the significant increase in Annexin V counts over time compared to OH14 or TRAIL treatment alone (Fig. 1a, b). This conformed to the changes observed when cFLIP was targeted by siRNA in PC-3M cells (Fig. 1c; Supplementary Fig. 1A). As an additional confirmation that the observed effects following cFLIP inhibition with OH14 were mediated through activation of apoptosis, each treatment arm was combined with the addition of a pan-caspase inhibitor (Z-VAD-FMK), leading to the complete rescue of OH14 mediated sensitization to TRAIL in PC-3M cells (Fig. 1d).

Finally, following each treatment viable cells were seeded into low-density colony forming assays without any additional exposure to treatments to measure changes in cancer stem/progenitor cell activity. In both LNCaP and PC-3M cell lines colony forming efficiency was significantly reduced in cells treated with OH14 and TRAIL compared to DMSO controls, which was not observed when each agent was treated as a monotherapy (Fig. 1e-g).

Together this data demonstrates the ability of the cFLIP inhibitor OH14 to sensitize prostate cancer cells and CSCs to TRAIL-mediated apoptosis.

### OH14 and TRAIL treatment inhibits colony formation in primary prostate cancer samples

Having shown the pro-apoptotic effects and anti-cancer stem/progenitor activity of cFLIP inhibition using OH14 in addition to TRAIL on established prostate cancer cell lines, we next sought to confirm these effects using models more closely related to prostate cancers observed clinically. A panel of primary patient-derived lines were generated from prostate tissues sampled from routine radical prostatectomy or transurethral resection of the prostate (TURP). The lines created were derived from benign prostatic hyperplasia (BPH) and prostate cancer samples ranging from Gleason grades 6-9. Each line was assessed for changes in tumour cell viability and colony formation following treatment.

**Fig. 2:**
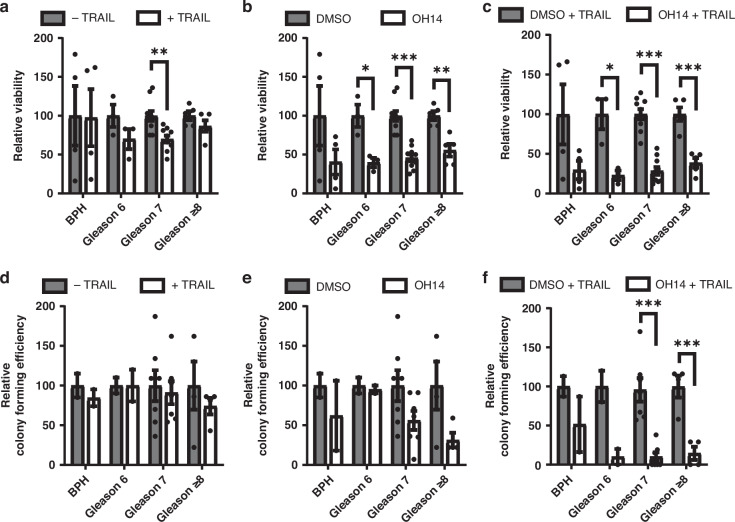
Investigating the response of primary prostate cell lines to OH14 and TRAIL. **a** Established primary prostate cell lines from BPH, Gleason 6, Gleason 7 or Gleason ≥8 samples were treated with TRAIL (20 ng/ml) for 18 h, **b** OH14 (100 µM) for 19 h or **c** OH14 for 1 h followed by TRAIL for 18 h before viability was determined by cell titre blue. Viable cells were counted and seeded at low density into colony formation assays following treatment with (**d**) TRAIL (**e**) OH14 or (**f**) OH14 and TRAIL. Colonies were manually counted and colony forming efficiency was plotted as a percentage of the number of cells initially seeded. Each data point is representative of the mean value from 3 biological repeats of an individual sample with error bars indicating SEM from all samples in that group. *p < 0.05, **p < 0.01, ***p < 0.001, two-tail unpaired t-test comparing treatment against control for each group.

Overall, OH14 had a greater effect on prostate tumour cell viability than TRAIL. The effect of TRAIL treatment alone on overall viability was minimal, with a small but significant reduction observed in Gleason 7 samples (Fig. 2a). In contrast, inhibition of cFLIP using OH14 alone significantly reduced viability in all cancer groups irrespective of grade (Fig. 2b). A similar trend was observed in BPH samples, however a high degree of variability meant that this was not significant (Fig. 2b). Combined treatment resulted in an even greater reduction in viability across each sample group compared to TRAIL treatment alone which was significant in each set of samples other than BPH (Fig. 2c).

Only 15 out of the 22 samples had the ability to form colonies following treatment when seeded at low density in the presence of STO feeder cells. In samples able to form colonies, TRAIL treatment alone had no effect on colony forming efficiency (Fig. 2d). cFLIP inhibition with OH14 resulted in an insignificant reduction in the colony forming ability of Gleason 7 and Gleason ≥8 samples, although when these groups were combined the reduction was significant (*p* = 0.008) (Fig. 2e). Combining TRAIL and OH14 treatments, resulted in a reduction in colony forming efficiency across each set of cancer samples, which was significant in both Gleason 7 and ≥8 groups (Fig. 2f).

This data confirms the ability of OH14 to sensitize prostate cancer to TRAIL mediated apoptosis and further highlights the effect of this combination on disrupting clonal expansion, indicative of CSC progenitor-like activity, particularly in more aggressive prostate cancers.

### Castrate-resistant prostate cancers exhibit elevated cFLIP levels

Our data suggests that cFLIP inhibition, particularly when in the presence of TRAIL could be a viable therapeutic strategy for targeting both bulk viability and CSCs, particularly in patients with high grade prostate cancers. The corollary to this conclusion is that high-grade late-stage prostate cancers might be expected to express high levels of cFLIP as a protection from innate tumour surveillance mechanisms. Indeed, the expression of cFLIP at the protein level is also known to be increased in CRPC compared to normal prostate tissue, while hormone therapy can suppress cFLIP expression [6]. Here we set out to test these assumptions using the Prostate Cancer Atlas to determine cFLIP mRNA levels in different prostate cancer molecular subtypes associated with tumour progression [27].

**Fig. 3:**
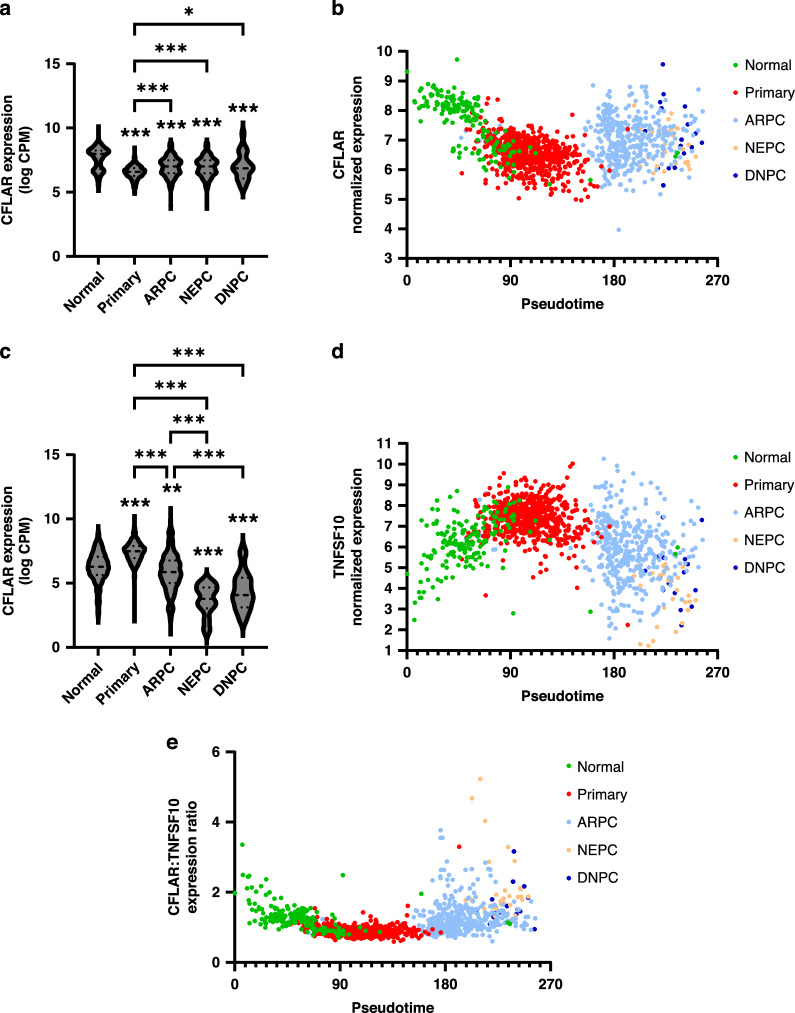
Analysing *CFLAR* and *TNFSF10* expression during prostate cancer progression. **a** The Prostate Cancer Atlas was used to analyse *CFLAR* expression in prostate tissue from normal (n = 173), primary (n = 708), ARPC (n = 428), NEPC (n = 34) and DNPC (n = 22) samples. **b** *CFLAR* expression from each group was compared against disease progression by plotting against inferred pseudotime. **c** The expression of *TNFSF10* was compared in the same dataset and (**d**) was also plotted to determine how expression changed during disease progression. **e** A ratio of *CFLAR* and *TNFSF10* expression was calculated and plotted against disease progression. Violin plots show data distribution with dashed lines highlighting the median and quartiles. *p < 0.05, **p < 0.01, ***p < 0.001, One-way ANOVA with Tukey correction for multiple comparisons.

In contrast to previously published protein analysis, cFLIP *(CFLAR)* expression was significantly lower in all cancer subtypes compared to normal prostate tissue, however expression was significantly increased in more aggressive, CRPC samples expressing androgen receptor (ARPC), with neuroendocrine features (NEPC) or negative for both androgen receptor and neuroendocrine features (DNPC) compared to primary tumours (Fig. 3a). This dynamic change in *CFLAR* expression was observed in pseudotime plots, which is an inferred model of tumour progression. This data demonstrates that although *CFLAR* expression was reduced in primary tumours compared to normal prostate tissue, it does increase in more advanced late-stage prostate cancers (Fig. 3b). This observation was supported by analysis of the TCGA dataset which also showed a significant reduction in *CFLAR* expression in primary prostate tumours compared to normal samples, and increased *CFLAR* expression correlated with higher Gleason grade (Supplementary Fig. 2A&B).

Conversely, TRAIL (*TNFSF10)* expression was significantly increased in primary tumour samples compared to normal, however in all CRPC subtypes *TNFSF10* was significantly reduced compared to both normal and primary prostate samples (Fig. 3c). Expression was lowest in NEPC and DNPC samples and significantly reduced compared to ARPC, suggesting that tumour-specific loss of *TNFSF10* expression correlates with progression to the most hard-to-treat subtypes of prostate cancer (Fig. 3d). Analysis of TCGA data again supported these observations (Supplementary Fig. 2C&D). Finally, the expression ratio of cFLIP versus TRAIL demonstrated a clear relationship with disease progression in CRPC samples (Fig. 3e).

Combined, these data suggest that cFLIP inhibition, with or without additional exogenous agents such as TRAIL, may be more effective in patients with CRPC and particularly patients that have developed NEPC or DNPC, for which there are currently very few therapeutic options.

### Combined treatment of OH14 and TRAIL inhibits tumour formation in vivo

Given the potential therapeutic benefit of inhibiting cFLIP, particularly for targeting colony forming capacity in later stage disease subtypes, we next wanted to determine the in vivo relevance of these in vitro observations in a model of late-stage hormone refractory disease. Although colony forming assays are an accepted surrogate for changes to cancer stem/progenitor activity, limiting dilution experiments in vivo remain the gold standard for assessing CSC activity [31]. Thus, in order to assess the effects of cFLIP inhibition with OH14 and TRAIL on CSC activity in an advanced prostate cancer setting, we investigated the effects of these treatments on a primary PDX model of CRPC by limiting dilution transplantation [26].

**Fig. 4:**
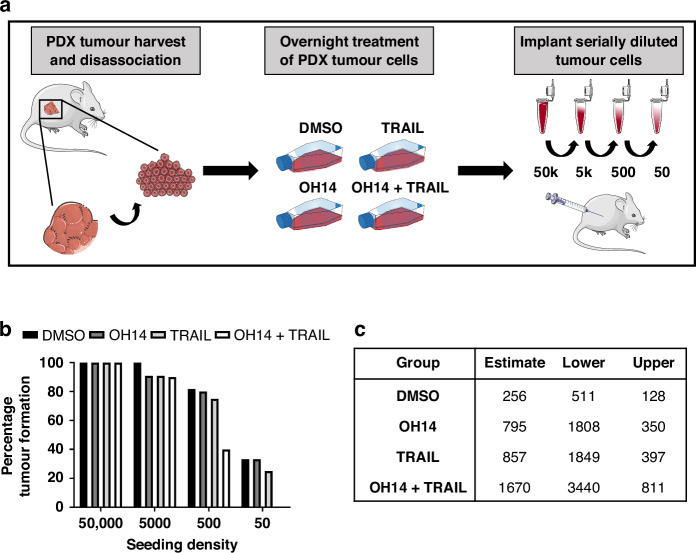
Determining the effect of OH14 and TRAIL treatment on CSC activity in vivo using a CRPC PDX model. **a** Graphical representation of the limiting dilution assay performed using H455 CRPC PDX cells. Briefly, PDX tumours were harvested, cells disaggregated and treated with DMSO (n = 34 total tumours implanted), OH14 (n = 33), TRAIL (n = 32) or combination (n = 28) for 24 hours prior to re-transplanting either 50,000, 5000, 500 or 50 cells into immunocompromised recipient mice (n = 18-22 total mice used per treatment group). **b** The percentage of tumours formed at each seeding density following each treatment regime was plotted before (**c**) limiting dilution analysis was performed to estimate the frequency of tumour initiating cells (CSCs), represented as the ratio of tumour cells per stem cell, with all data points included in the analysis.

H455 PDX tumours were generated in immunocompromised NSG mice before being harvested, disassociated into single cells and treated in suspension for 24 h with either DMSO, OH14, TRAIL or the combination treatment before viable cells were re-transplanted at various seeding densities and monitored for 3 months for tumour formation (Fig. 4a). At the lower seeding densities of 500 and 50 cells tumour formation rates were similar in control and single treatment groups (500 cells: DMSO = 82%, OH14 = 80%, TRAIL = 75% and 50 cells: DMSO = 33%, OH14 = 33%, TRAIL = 25%). However, in the combination treated group only 40% of mice formed tumours when 500 cells were seeded, and no tumours were observed at the lowest seeding density (Fig. 4b). Calculating the estimated frequency of tumour initiating cells within each treatment group revealed that less than one sixth of the tumour initiating (CSCs) remained viable following combination treatment (1 in 1670 cells) compared to vehicle treatment control (1 in 256 cells), while less than one third of the CSCs remained after OH14 or TRAIL treatment alone (1 in 795 and 857 cells respectively) (Fig. 4c). This data confirms that cFLIP inhibition using OH14, particularly in combination with TRAIL treatment significantly impacts prostate CSC activity in a model of CRPC.

### Combined OH14 and docetaxel treatment reduces CRPC tumour growth

Despite the beneficial effects observed with combined OH14 and TRAIL in both established and primary cell lines as well as the H455-primary model of CRPC, current standard of care approaches for the treatment of CRPC would mandate the use of novel targeting strategies alongside chemotherapy such as docetaxel. Indeed, suppression of cFLIP by siRNA has previously been demonstrated to sensitise PC-3 and LNCaP cells to docetaxel in vitro [14]. To explore this further and to confirm the in vivo relevance of these findings for tumour efficacy, we sought to determine the relative contribution of OH14 and docetaxel in the H455 CRPC PDX model in vivo.

**Fig. 5:**
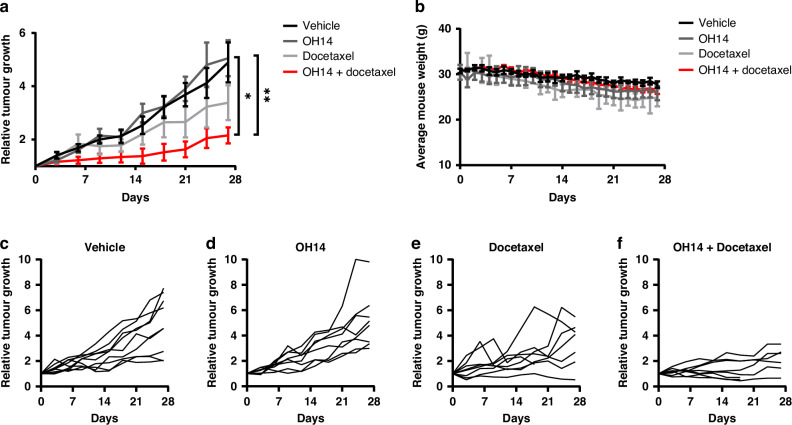
Determining the response of OH14 and docetaxel treatment in CRPC PDX xenografts. **a** H445 CRPC PDX xenograft tumours between 20-100mm^3^ were treated with either vehicle (n = 9 tumours), OH14 (10 mg/kg, i.p, daily 5 a week) (n = 8), docetaxel (10mk/kg, i.p, once a week) (n = 8) or OH14 and docetaxel (n = 8) for 4 weeks (n = 6 total mice used per treatment group). Tumours were measured using callipers and relative tumour volume calculated by normalizing tumour volumes to their initial size before treatment. **b** Mouse weights were recorded daily. **c**–**f** Individual tumours were plotted to demonstrate the effect of each treatment clearly. *p < 0.05, **p < 0.01, Two-way ANOVA with Tukey correction for multiple comparisons. No tumours were excluded from the analysis.

H455 PDX tumours were allowed to establish prior to treatment with a combination of OH14 and/or docetaxel over a 4-week period. In contrast to the previous in vitro findings, cFLIP inhibition with OH14 had no effect on tumour growth compared to vehicle treated tumours (Fig. 5a, c and d; Supplementary Fig. 3A), and while docetaxel treatment alone reduced tumour growth, this was also not significant (Fig. 5a and e). However, the combination of OH14 augmented the docetaxel response, resulting in a 70% reduction in tumour burden compared to vehicle control (Fig. 5a and f). This provided, for the first time, in vivo evidence to support the previously published in vitro data demonstrating synergy between these two interventions. Although one mouse in the combination group had to be culled early due to diarrhoea all mouse weights remained stable across all treatment groups, indicating that this combination treatment was safe to administer (Fig. 5b).

### Inhibition of cFLIP using OH14 sensitises docetaxel resistant PC-3 cells to TRAIL-mediated cell death

**Fig. 6:**
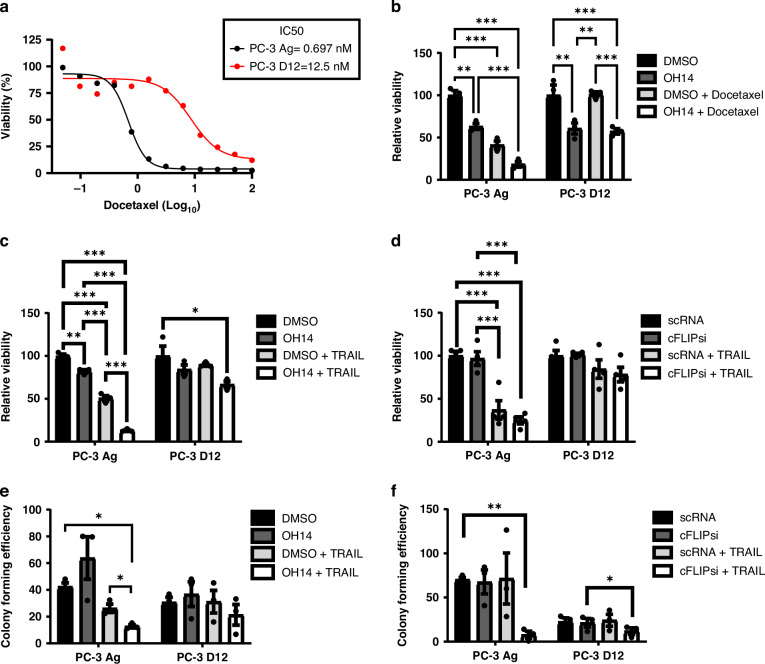
Assessing the response of docetaxel resistant PC-3 cells to OH14 plus TRAIL or docetaxel treatment. **a** Docetaxel resistant PC-3 cells (PC-3 D12) and age matched controls (PC-3 Ag) were treated with docetaxel ranging from 0.05-100 nM for 72 h before viability was determined by cell titre blue. Data shown is representative of 3 independent experiments with IC50 values calculated on the average best-fit value. **b** Both cell lines were treated with OH14 (100 µM) and/or docetaxel (1 nM) for 72 h and viability was assessed by cell titre blue assay. Each cell line was treated with (**c**) OH14 for 1 h or (**d**) cFLIP siRNA for 24 h followed by TRAIL (20 ng/ml) for 18 h and assessed for changes in viability. Following treatment with (**e**) OH14 or (**f**) cFLIP siRNA +/- TRAIL, viable cells were counted and seeded at low density into colony formation assays. Each data point represents a biological repeat with error bars shown as SEM of all data points. * p < 0.05, ** p < 0.01, *** p < 0.001, One-way ANOVA for endpoint comparisons or Two-way ANOVA for time course Incucyte assays, both with Tukey correction for multiple comparisons.

Given the promising data suggesting that cFLIP inhibition combined with docetaxel could significantly impact overall viability and tumour efficacy in high grade CRPC, we next sought to determine whether this strategy could be beneficial to men with aggressive docetaxel resistant prostate cancer. Docetaxel is one of the very few treatment options currently available for men with mCRPC, however resistance often occurs within 3 years of treatment [32, 33]. Therefore, new therapeutic strategies that can improve initial responses or resensitize patients to chemotherapy are urgently needed, while alternative therapeutic options for docetaxel resistant prostate cancer need to be explored. Hence, we looked to test whether the pharmacological inhibition of cFLIP by OH14 in combination with either docetaxel or TRAIL treatment impacted on prostate cancer cell, and CSC viability in a model of docetaxel acquired resistance (PC-3 D12) [22] (Fig. 6a).

Inhibition of cFLIP using OH14 alone for 72 hours reduced the viability of both PC-3 D12 cells and age matched control PC-3 cells (PC-3 Ag) (Fig. 6b), while, as expected, docetaxel treatment alone only reduced viability in PC-3 Ag cells (Fig. 6b). Combining both treatments further reduced the viability of PC-3 Ag cells but did not improve the response of PC-3 D12 cells to docetaxel. This confirmed that targeting cFLIP could be a promising therapeutic approach in docetaxel sensitive prostate cancer and that prolonged OH14 treatment could also be used to target docetaxel resistant prostate cancer.

We next sought to determine whether docetaxel resistance impacted on the previously observed responses of PC-3 cells to TRAIL (Fig. 1). After 24 hours of treatment PC-3 D12 cells were much more resistant to TRAIL or OH14 monotherapies compared to the age-matched PC-3 Ag cells, however the combination of the two did result in a diminished yet significant reduction in viability compared to vehicle treated controls (Fig. 6c). These experiments were replicated with cFLIP siRNA in place of OH14, where similar trends were observed (Fig. 6d). The same was true for colony forming ability, whereby PC-3 D12 cells exhibited diminished responses to combined TRAIL and cFLIP inhibition, and no response to either treatment alone (Fig. 6e, f).

Together this data suggests that acquired docetaxel resistance imparts a concomitant TRAIL resistance in prostate cancer cells, while targeting cFLIP in combination exhibits a marginal improvement to either treatment irrespective of docetaxel resistance status.

## Discussion

Given the poor outlook for patients diagnosed with advanced prostate cancer, which is largely due to intrinsic or quickly acquired resistance to the currently available therapies, there is a continued need to pursue new therapeutic avenues. Genetic suppression of cFLIP in combination with TRAIL or chemotherapy has been shown to be an effective approach to kill prostate cancer cells through the induction of apoptosis [6, 13–15], while cFLIP inhibition and TRAIL treatment can also target CSCs in breast cancer [17]. In this study we tested a novel cFLIP inhibitor, OH14, to determine whether prostate CSCs can also be targeted by this combination treatment and whether OH14 could also be used to sensitise prostate cancer cells to docetaxel.

Combined treatment of OH14 and TRAIL was tested in a range of established and primary prostate cancer cell lines and reduced overall tumour cell viability as well as CSC activity. This reduction in CSC activity following combined treatment was confirmed by limiting dilution assessment in vivo using a CRPC PDX model, which could have important clinical implications as the search continues for therapies that are able to target persister cells that are resistant to treatment and provide long-lasting benefits to patients. This could be particularly pertinent in hard-to-treat NEPC and DNPC subtypes, where *CFLAR* expression was shown to be significantly upregulated compared to HSPC. In addition, we also showed that cFLIP inhibition with OH14 could be used in combination with docetaxel to enhance its anti-tumour effects and re-sensitise docetaxel resistant prostate cancer cells.

These findings are in line with previous studies demonstrating the pro-apoptotic effects of genetically silencing cFLIP or TRAIL treatment alone in prostate cancer cells and the significantly enhanced anti-cancer effects of combining these two approaches [6, 13–15]. In addition, silencing cFLIP expression has been shown to improve the efficacy of docetaxel, which we now confirm with pharmacological inhibition of cFLIP [14]. Interestingly, the combination of docetaxel and TRAIL has been shown to work synergistically to reduce bulk viability in prostate cancer cell lines but has little effect on the CSC population which remain viable after treatment [34**–**36]. Given our data demonstrating the ability of OH14 and TRAIL to target prostate CSC’s, targeting cFLIP in combination with docetaxel and TRAIL could further improve the sensitization of both bulk and CSC populations to reduce the potential of tumours relapsing.

Although several preclinical studies have demonstrated the potent anti-cancer effects of inhibiting cFLIP, this approach is yet to be tested clinically. The development of new small molecule modulators, which are able to sensitize cancer cells to TRAIL-mediated apoptosis, offer new hope that this approach could be tested in patients in the future [19**–**21, 37]. Further refinements are required to increase the potency of these inhibitors at lower nanomolar concentrations before they can be taken forward to clinical investigation, however, both MDM2 and histone deacetylase (HDAC) inhibitors, have been shown to suppress cFLIP expression indirectly to induce apoptosis in prostate cancer cells [6, 38]. Given that these approaches have already been tested clinically in prostate cancer [39, 40], they could be used as a faster route to clinic to target prostate cancers where cFLIP is overexpressed, however these will likely need to be used alongside additional treatments such as TRAIL or chemotherapy to improve potency. Despite its limited efficacy in a phase III clinical study, Dulanermin, a human recombinant soluble TRAIL treatment, demonstrated a low toxicity profile even when administered alongside chemotherapy, suggesting that it is feasible for TRAIL treatment to be used alongside indirect targeting of cFLIP [41].

Despite observing increased *CFLAR* expression in hard-to-treat NEPC and DNPC samples compared to hormone-sensitive prostate cancers, we also observed a significant reduction in *CFLAR* expression in all prostate cancer subtypes compared to normal prostate tissue, contradicting previous work investigating cFLIP at the protein level [6]. Posttranscriptional regulation of cFLIP is known to be controlled though both mTOR dependent and independent pathways which could account for the differences between cFLIP mRNA and protein levels [42**–**44]. Loss of USP8/AIP4 mediated ubiquitination of cFLIP as a result of increased pAKT could also explain these discrepancies, particularly given that pAKT is commonly upregulated through PTEN loss, particularly in late-stage prostate cancers [45, 46]. Although the relationship between cFLIP and PTEN/PI3K/AKT signalling has not been extensively investigated in prostate cancer, further studies testing cFLIP inhibition and/or TRAIL treatment in combination with PI3K/AKT pathway inhibitors could be an interesting therapeutic avenue to explore. Whether cFLIP inhibition with OH14 is also able to sensitize prostate tumours to other treatments such as AR-targeting agents has not been explored here, however given that siRNA-mediated silencing of cFLIP has been shown to improve prostate cancer response to bicalutamide, this could also be an interesting avenue to investigate in the future [6].

Interestingly, rodent model studies have shown that cFLIP can be upregulated by androgens to protect against TRAIL-mediated apoptosis, while cFLIP loss is critical for castration-induced apoptosis and upregulation of cFLIP can inhibit this process resulting in progression to androgen-resistance [47, 48]. Our RNA-seq analysis suggests that there may be a switch during the transition to CRPC whereby *CFLAR* becomes upregulated, indicating that cFLIP inhibition may not be appropriate for low grade, hormone sensitive primary tumours that express low levels of cFLIP. Upregulation of *CFLAR* correlated with a loss of *TNFSF10* in more advanced prostate cancer samples, in line with previous work indicating that high *TNFSF10* expression is associated with low-grade prostate cancer and good prognosis [49, 50]. This suggests that a combination of cFLIP inhibition and TRAIL treatment may be highly effective against late-stage NEPCs, which have significant disruption to the extrinsic apoptosis pathway. Given that NEPC tumours currently lack any form of targeted therapies and are emerging at an increased frequency due to the widespread use of AR-targeting therapies, exploring this therapeutic approach could have important clinical implications for these hard-to-treat cancers in the future [51, 52]. Further investigation is required to confirm this hypothesis, especially given that a large proportion of patients with NEPC will have received chemotherapy and our data suggests that acquired docetaxel resistance conveys cross-resistance to TRAIL.

In summary, the data presented here demonstrates the potential of small molecule cFLIP modulators to be used in combination with TRAIL treatment to kill prostate cancer cells and specifically target the hard-to-treat CSC population. Direct inhibition of cFLIP is also able to improve the sensitivity of prostate cancer cells to docetaxel, which could be an interesting approach for treating late-stage NEPCs that express high levels of *CFLAR*.

## Supplementary information

Supplementary Figure 1

Supplementary Figure 2

Supplementary Figure 3

## Data Availability

All data generated in this study is stored locally on password protected archive servers at the University of the West of England and is available on request to the corresponding author.
