# Supplementary figures and images for: Inhibition of c-FLIP alongside TRAIL treatment suppresses prostate cancer stem cell activity
Source: Br J Cancer. 2026 Mar 3;134(9):1300–10. doi: 10.1038/s41416-026-03359-4 (PMC13079851; doi:10.1038/s41416-026-03359-4)

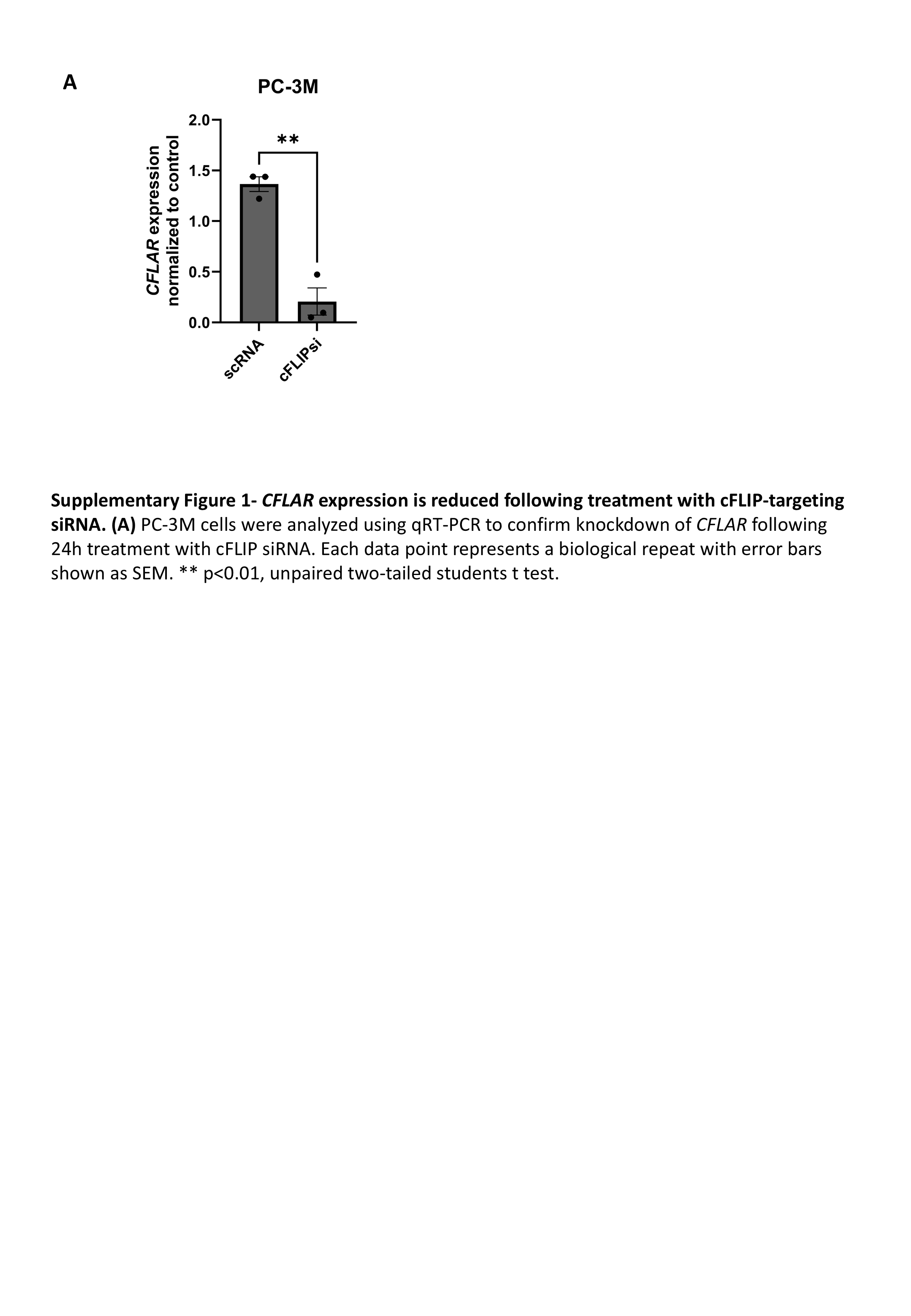

Supplement: Supplementary file 1 — Supplementary Figure 1 [file 41416_2026_3359_MOESM1_ESM.tif]

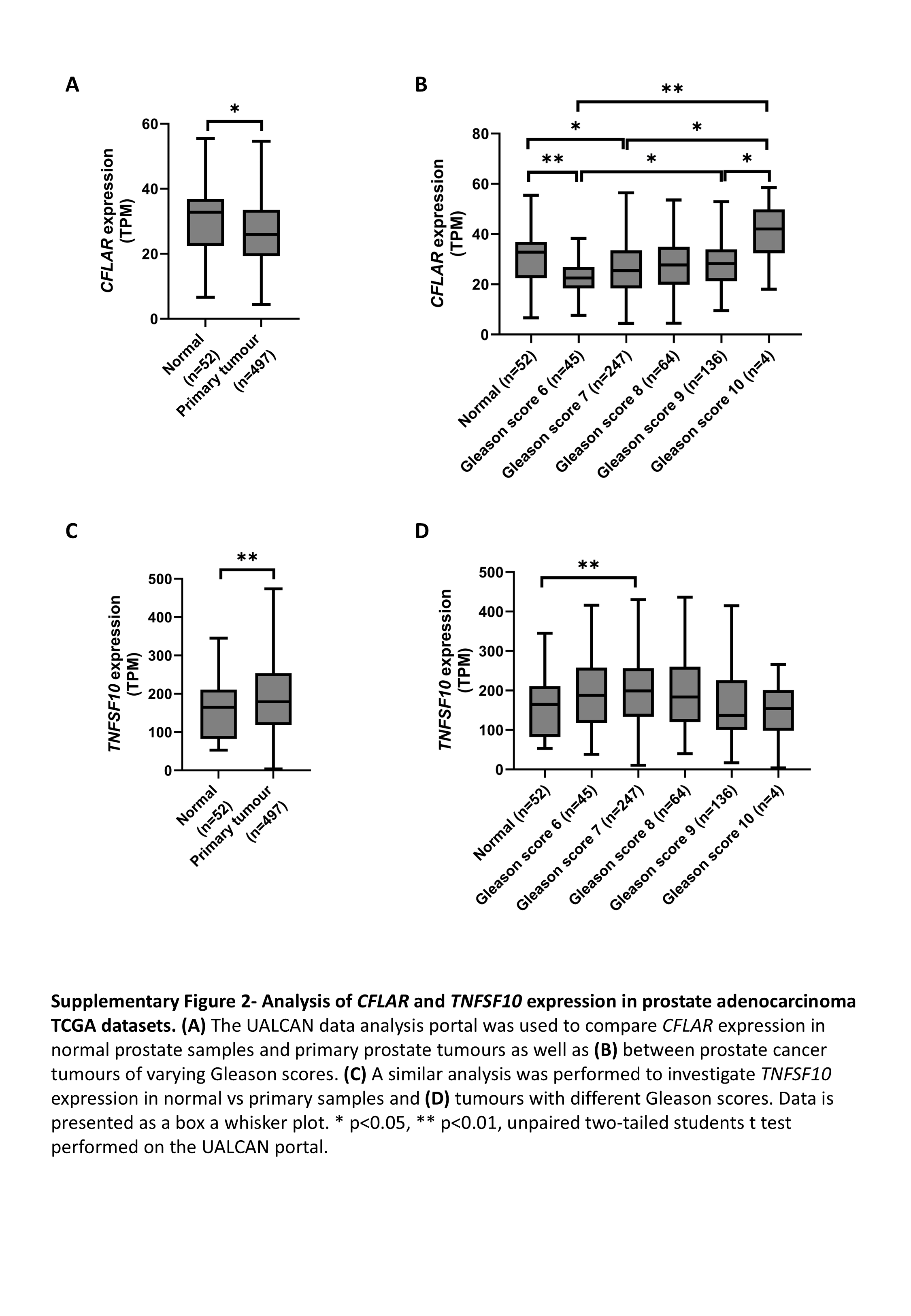

Supplement: Supplementary file 2 — Supplementary Figure 2 [file 41416_2026_3359_MOESM2_ESM.tif]

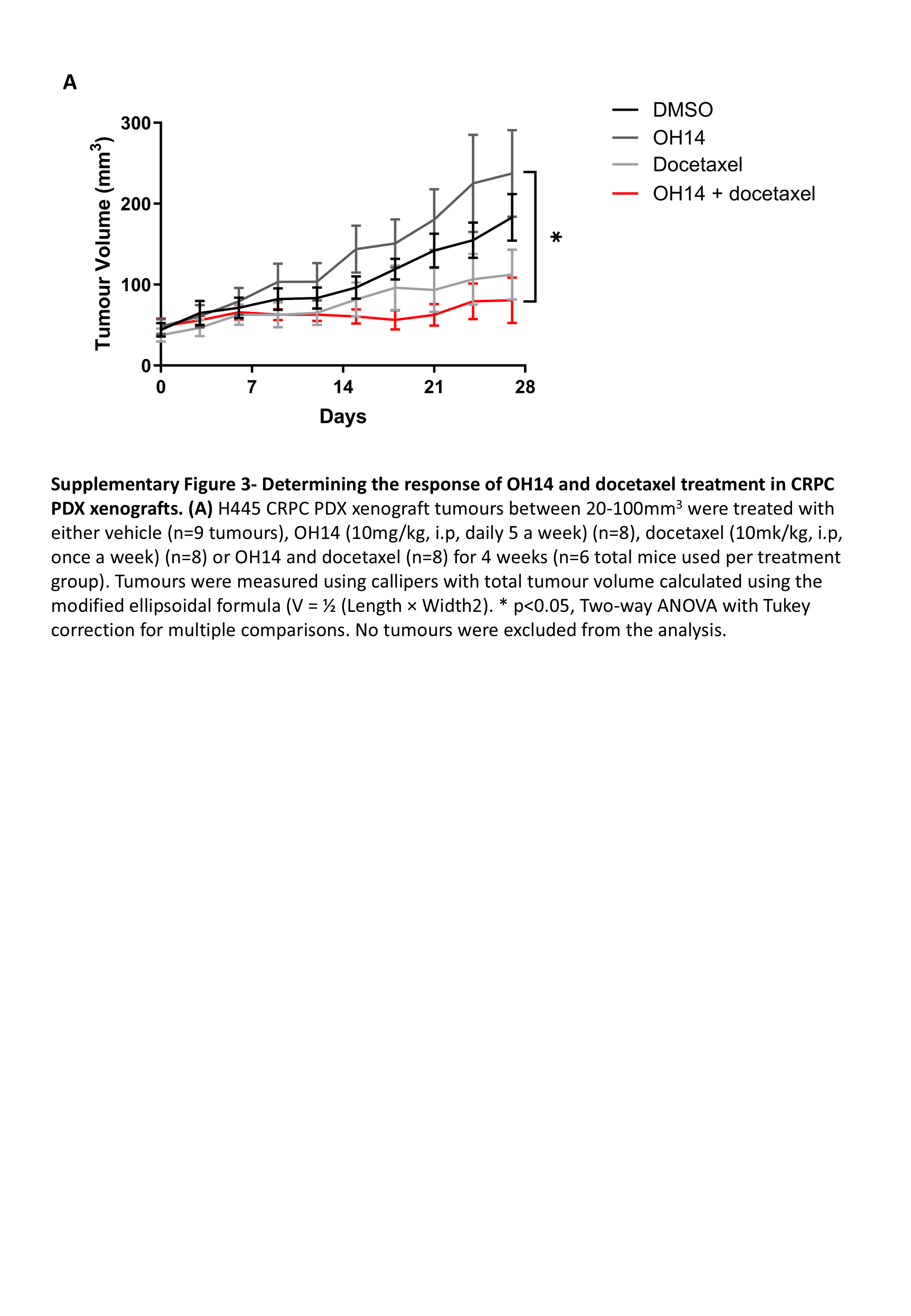

Supplement: Supplementary file 3 — Supplementary Figure 3 [file 41416_2026_3359_MOESM3_ESM.tif]
